# Supplementary material for: Recombinant Anti-PF4 Antibodies Derived from Patients with Vaccine-Induced Immune Thrombocytopenia and Thrombosis (VITT) Facilitate Research and Laboratory Diagnosis of VITT
Source: Vaccines (Basel). 2024 Dec 24;13(1):3. doi: 10.3390/vaccines13010003 (PMC11769302; doi:10.3390/vaccines13010003)
Supplement: Supplementary file 1 [file vaccines-13-00003-s001.zip › vaccines-3344177-supplementary.pdf]

SUPPLEMENTARY MATERIAL - Recombinant anti-PF4 antibodies derived from patients with vaccine-induced immune thrombocytopenia and thrombosis (VITT) facilitate research and laboratory diagnosis of VITT

Luisa Müller [1*], Venkata A. S. Dabbiru [1*], Lucy Rutten [2], Rinke Bos [2], Roland Zahn [2], Stefan Handtke [1], Thomas Thiele [1], Tom Paul Gordon [3], Andreas Greinacher [1], Jing Jing Wang [3#], and Linda Schönborn [1#§]

* equally contributed as first authors, # equally contributed as senior authors

1 Institut für Transfusionsmedizin, Universitätsmedizin Greifswald, Greifswald, Germany; Luisa.Mueller@med.uni-greifswald.de, Venkata.Dabbiru@med.uni-greifswald.de, Stefan.Handtke@med.uni-greifswald.de, Thomas.Thiele@med.uni-greifswald.de, Andreas.Greinacher@med.uni-greifswald.de

2 Janssen Vaccines & Prevention BV, Leiden, the Netherlands.; LRutten@ITS.JNJ.com, RBos6@its.jnj.com, RZahn@its.jnj.com

3 Department of Immunology, College of Medicine and Public Health, Flinders University and SA Pathology, Bedford Park, SA, Australia; T.Gordon@flinders.edu.au, Jingjing.Wang@flinders.edu.au

§ Correspondence: Linda.Schoenborn@med.uni-greifswald.de, Tel.: +49 3834/86 5479

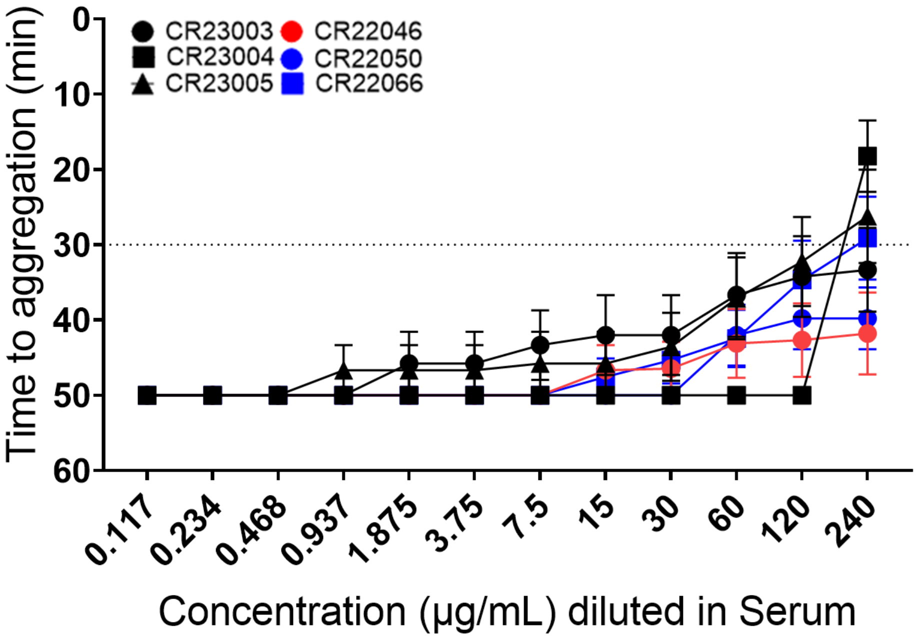

Supplementary Figure S1: Functional platelet activation control assays (PIPA test without added PF4 =buffer control) using recombinant antibodies (rAbs) at concentrations between 0.117-240µg/ml. Cut-off at 30 min of the test is represented by the dashed line, platelets that were not activated within the 45 min observation time were assigned to 50 min (=no aggregation). Tests were carried out using 9 independent platelet donors. Results are given as mean ±SEM. For concentrations at which not all platelets aggregated, means are calculated using the assigned 50 min for no aggregation.

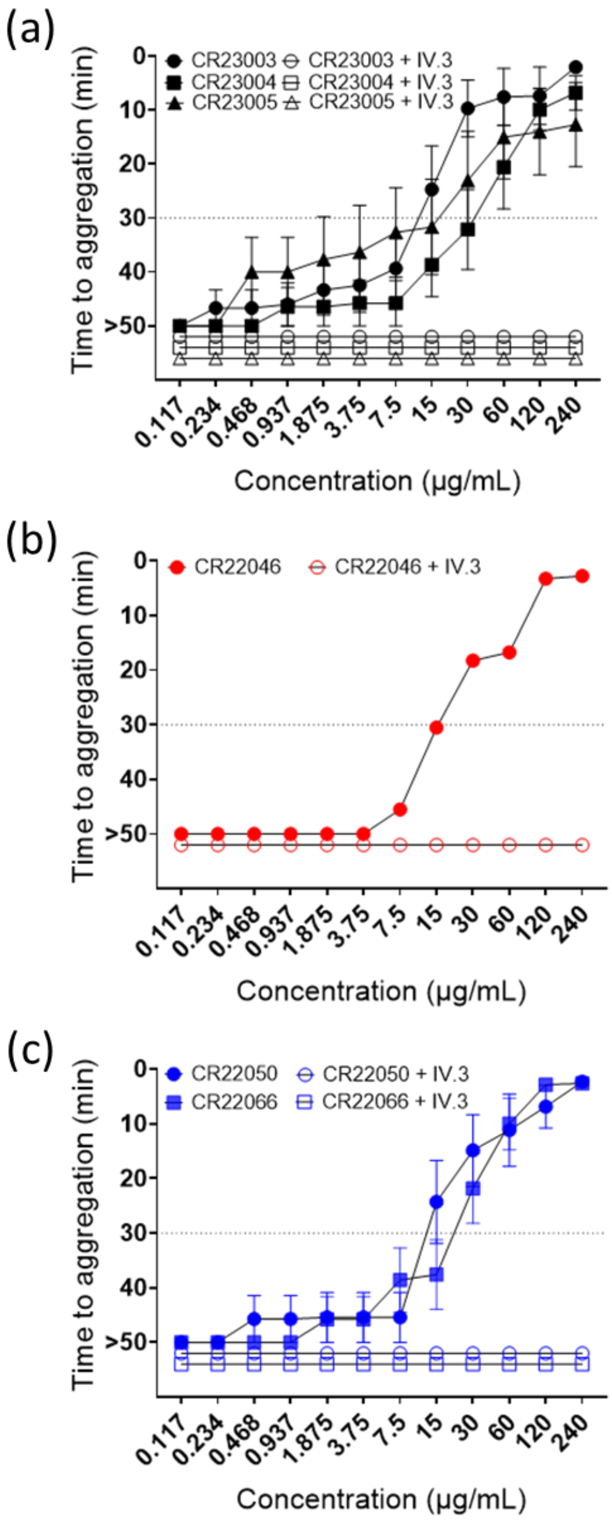

Supplementary Figure S2: Functional platelet activation assays (PIPA test) using recombinant antibodies (rAbs) at concentrations between 0.117-240 µg/ml of (a) CR23003-CR23005, (b) CR22046, (c) CR22050 and CR22066. Cut-off at 30 min of the test is represented by the dashed line, platelets that were not activated within the 35 min observation time were assigned to 50 min (=no aggregation). FcγRIIa-dependency of platelet activation was determined by mAb IV.3 that blocks FcγRIIa-mediated platelet activation. Throughout all tests IV.3-treated samples did not aggregate. For better visualization data points assigned to no aggregation are stacked. Tests were carried out using 9 independent platelet donors (mAbs) or 4 independent donors (mAbs + IV.3), results are given as mean±SEM.
